# Plant Stress Scenarios Differentially Affect Expression and IgE Reactivity of Grass Group-1 Allergen (β-Expansin) in Maize and Rice Pollen

**DOI:** 10.3389/falgy.2022.807387

**Published:** 2022-02-10

**Authors:** Yotin Juprasong, Wisuwat Songnuan

**Affiliations:** ^1^Graduate Program in Toxicology, Faculty of Science, Mahidol University, Bangkok, Thailand; ^2^Center of Excellence on Environmental Health and Toxicology, Faculty of Science, Mahidol University, Bangkok, Thailand; ^3^Systems Biology of Diseases Research Unit, Faculty of Science, Mahidol University, Bangkok, Thailand; ^4^Department of Plant Science, Faculty of Science, Mahidol University, Bangkok, Thailand

**Keywords:** grass pollen, pollen allergy, qRT-PCR, environmental fluctuation, tropical/subtropical crop plants

## Abstract

Grass pollen is among the most common outdoor aeroallergens eliciting pollen allergies throughout the world. Grass group-1 allergen or β-expansin is recognized as a major pollen allergen, particularly in the grass family Poaceae. Expression of β-expansin has been shown to be dynamic and can be influenced by environmental stresses. This study evaluated the relative expression of β-expansin and IgE-binding ability of crude pollen extract protein of rice and maize under three different stress conditions: flood, salt, and drought. After 1 week of treatments, anthers containing pollen were collected followed by RNA extraction and cDNA synthesis. To evaluate relative expression, qRT-PCR was performed using specific primers for β-expansin and reference genes. Physiological characteristics of treated and untreated maize and rice: plant height; fresh weight of anthers; number of inflorescences, anthers, and pollen grains were also recorded. To assess IgE-binding ability of proteins in rice pollen extracts, soluble crude proteins were extracted and IgE immunoblot and ELISA were performed using serum samples from grass-allergic subjects and healthy control donors. Results showed that plant height, fresh weight of anthers, number of inflorescences, anthers, and pollen grains of both maize and rice decreased significantly under drought stress conditions, but not in other conditions. Expression of β-expansin in pollen of rice showed an apparent increase in all stress treatments relative to control samples. In contrast, a significant decrease of β-expansin expression was detected in maize pollen under all stress-treated conditions. IgE-reactive protein bands from rice pollen extract proteins were ~30 kDa, as expected of the grass-group 1 protein. The intensity of IgE-reactive protein bands and the level of IgE to rice pollen proteins showed significant differences among stress conditions. In conclusion, environmental stresses—flood, salt, and drought, can elicit a change of β-expansin expression and IgE reactivity to grass group-1 pollen allergens. Changes in expression level of this gene likely reflected its importance during stress. However, the response is highly dependent on different schemes employed by each plant species.

## Introduction

Atmospheric allergens are significant causes of allergic diseases of both the upper and lower respiratory tracts. Environmental fluctuations including global climate change affect the quantity, quality, and distribution of aeroallergens such as pollen. This phenomenon impacts the severity of seasonal allergic rhinitis and other seasonal allergic diseases worldwide (1–3).

Among the various airborne pollen types, grass pollen is considerably the most dominant type and the major allergenic source of outdoor aeroallergens globally (4). Major group-1 pollen allergenic protein is abundantly found in all grass species including tropical/subtropical allergenic grasses (5–10). For instance, Cyn d 1, Sor h 1, Pas n 1, Lol p 1, and Zea m 1 are recognized as major group-1 grass pollen allergens produced from Bermuda grass (*Cynodon dactylon*), Johnson grass (*Sorghum halepense*), Bahia grass (*Paspalum notatum*), ryegrass (*Lolium perenne*), and maize (*Zea mays*), respectively.

Previous reports showed that up to 90% of the grass-pollen allergic patients are sensitized to the grass group-1 allergen (11–13). Remarkably, the grass group-1 allergen was also reported as a major cross-reactive protein for allergic patients in Thailand. Thai grass-pollen allergic patients had high sIgE cross-reactivity in subtropical allergenic grasses such as Bermuda, Johnson, and Para grasses. Immunoblot analysis of pollen extracts from these three grass species demonstrated sIgE binding to the protein at 29–30 kDa that was identified as group-1 allergen, functionally characterized as β-expansin protein (14).

In general, allergenicity of pollen mostly depends on the sIgE reactivity to allergenic proteins contained within the pollen, as well as the expression levels of all allergenic proteins (15). The amounts of allergens, their quality and distribution can fluctuate and are often influenced by environmental factors (16–19). For instance, rising temperature and atmospheric CO_2_ concentration can induce plants to grow more vigorously, prolong the growing seasons and speed up flower development. These phenomena lead to pollen season shifting earlier as well as higher pollen production (20–24).

Apart from the influences of temperature and ambient CO_2_ level, other abiotic stresses such as flood, salinity, and drought also have impacts on plant allergens, particularly in terms of transcription levels and protein expressions (25). Plants have evolved several physiological and biochemical homeostatic balances to attain tolerance and adaptation to such stressful conditions by means of modulating gene expression, post-transcriptional RNA modification, or protein modification (26, 27). For example, teff (*Eragrostis tef*), a grass species known as a major crop in Ethiopia and also found abundantly in the Horn of Africa, was shown to have a significant upregulation of several genes related to either cell growth or stress responses such as β-expansins against waterlogging (28). Expansin-like B protein, a member of the expansin superfamily, was a notable 27-times higher in root tips of soybean seedlings after flooding compared to control samples (29). The peanut (*Arachis hypogaea*) pathogenesis-related class 10 (PR10) allergen transcript, namely *AhSIPR10*, was rapidly upregulated in callus cultures under saline conditions (30). Likewise, the PR10 proteins as well as several proteins with similarities to the PR10 family members found in peanut callus cultures were upregulated under salt stress (31). The analysis of expressed sequence tags in ragweed under drought stress showed increased expression of allergenic ragweed (*Ambrosia artemisiifolia*) proteins, especially Amb a 1, the major allergenic protein in pollen (32, 33).

Conversely, a study in buckwheat showed that prolamins and gliadins, the major allergens in cereal grains, were decreased under a drought-stress condition (34). Interestingly, well-known fruit allergens chitinases and thaumatin-like proteins had comparable levels in grapes (*Vitis vinifera*) treated with or without water stress, suggesting that these proteins may not be strongly influenced by environmental conditions (35). Apart from the effects of abiotic stresses on either plant transcript or protein expression, average pollen grains per anther and pollen fertility of drought-treated plants generally show remarkable decline (36).

Environmental fluctuations not only affect plants, but also increase the incidence of allergies worldwide (2, 22, 37). A 27-year study of airborne pollen dynamics in Italy demonstrated an obvious positive association between an increase in total pollen of various plant species such as olive and cypress by ~25% on average and a constant rise in temperature and radiation. Remarkably, these changes also showed a strong-positive correlation with allergic sensitization rates, suggesting that climate variations have a direct role in the epidemiologic impact of pollen allergy (38). In Thailand, as of 2019, the prevalence of allergic rhinitis in Thai children was slightly higher than the average of the Asia-Pacific and global prevalence. Concerningly, the allergic prevalence among Thai citizens has been rising continuously (21, 39, 40), although not enough evidence can directly pinpoint environmental changes as the culprit.

Although several studies have revealed the effects of plant abiotic stresses on pollen production and allergen transcript/protein expression levels, no information is available on the influences of abiotic stresses on the grass group-1 allergen (β-expansin) of major crop plants, particularly in the region of Southeast Asia. Therefore, this study aims to investigate the effects of abiotic stress conditions: flood, salinity, and drought, on the grass group-1 allergen transcript expression in rice (*Oryza sativa*) and maize (*Zea mays*). Furthermore, IgE binding to crude protein extracts was assessed to determine the change in immunoreactivity of rice pollen after stress treatments.

## Materials and Methods

### Plant Materials and Growth Condition

**Figure 1:**
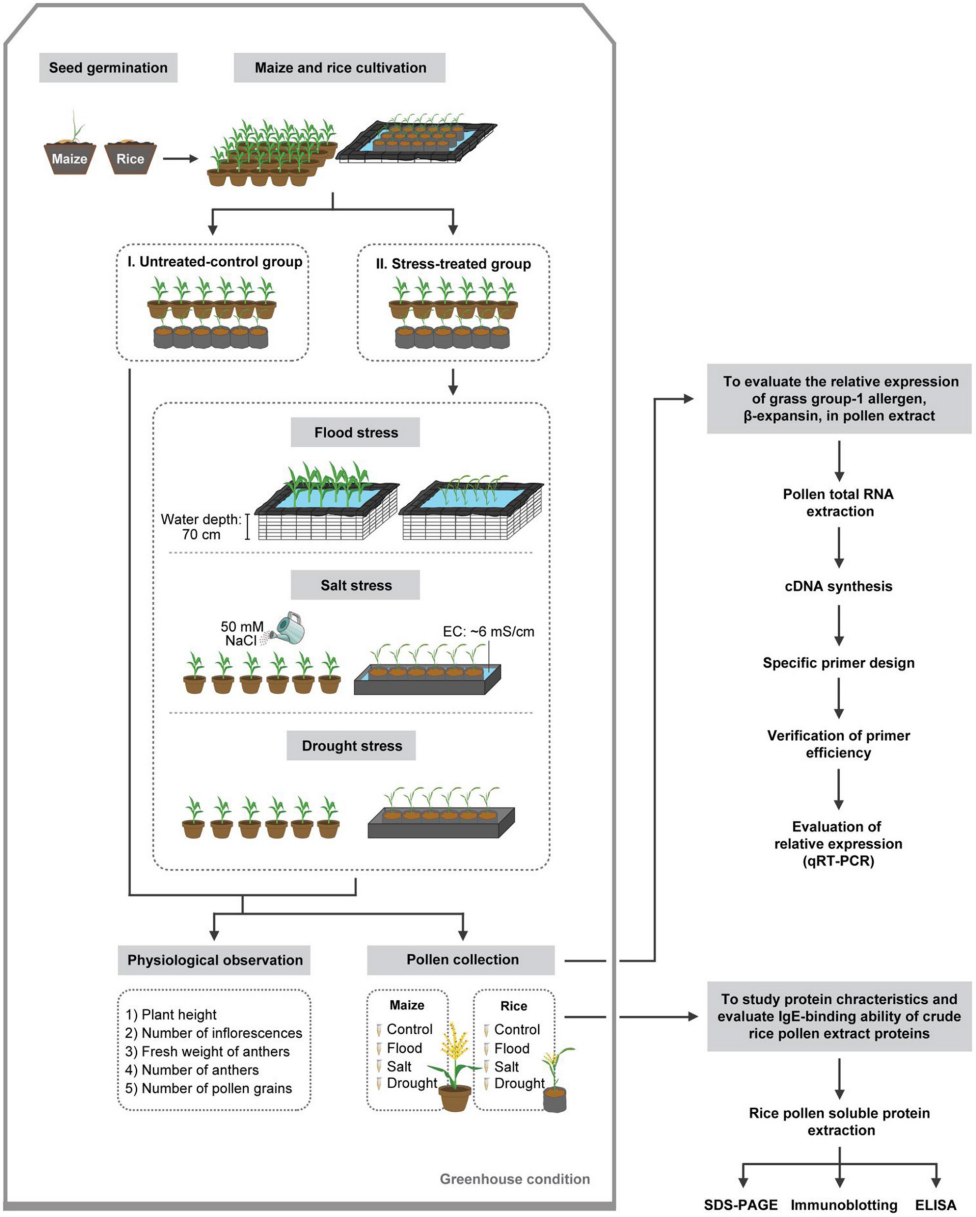
Schematic overview of experimental methods.

Rice (*Oryza sativa*) cultivar “Supan Buri 9” and maize (*Zea mays*) cultivar “Top Sweet 801” were chosen as representatives of the grass family Poaceae in this study. Rice and maize seeds imbibed tap water for 24 and 48 h, respectively. The imbibed seeds were germinated in a 50-well nursery tray containing soil to facilitate normal root and shoot developments for 14 days in a greenhouse. The 14-day old rice and maize seedlings were transplanted and grown in individual 5-L and 20-L pots, respectively. Fertilizer (46–0–0) was applied to the soil at 0.5 grams per pot before transplantation. Transplanted rice and maize plants were kept in a greenhouse. The potted rice plants were arranged in 231 × 273 × 28 cm plots built from cement blocks lined with double sheets of black polyester (PE) plastic sheet. Tap water was added into the plots until the water was 25 cm deep for irrigation, mimicking the rice cultivation condition in the field. The individually potted maize plants were irrigated every day. Experiments were conducted in a completely randomized block design with six replications (*n* = 6) per treatment: untreated control, flood-, salt-, and drought-treated conditions, with one plant per replicate (Figure 1). Each rice plant consisted of 5–8 culms, which matured at different time. All experiments were repeated three times independently, except those involving patient serum, which were performed only once due to serum sample limitation.

### Stress Treatments

Rice and maize plants were randomly divided into untreated control and stress-treated groups. Stress treatments include flood, salt, and drought. Stress treatments were applied to the plants as soon as their flag leaves became visible. The duration of all treatments was seven days, before pollen samples were collected.

#### Flood Stress

To simulate the flooding conditions, potted plants were moved to the experimental plots lined with black PE plastic sheets (155 × 156 × 84 cm for rice; 194 × 234 × 84 cm for maize). The plots were filled with tap water to 70 cm in depth, which was ~50 cm above the crown for rice and 40 cm for maize.

#### Salt Stress

Cultivated plants were subjected to 50 mM NaCl (Electrical conductivity, EC ~6 mS/cm) as salt stress treatment. For salt stress treatment of rice, water was completely drained out of the experimental plot and replaced with 50 mM NaCl solution to 25 cm depth. The EC was measured using an EC meter (LAQUAtwin EC-11, Horiba Ltd., Kyoto, Japan), and adjusted daily to 5.9–6.2 mS/cm. For salt stress treatment of maize, 500 ml of 50 mM NaCl solution was used for irrigation daily at 8 am.

#### Drought Stress

For rice, water was completely drained out of the plot to simulate the drought stress condition. Drought stress was imposed to maize by withholding water. For each treatment, normally irrigated plants were used as controls.

### Physiological Observation and Pollen Collection

After seven days of treatments, all plants of each condition were in a flowering phenological stage. Plant height, number of inflorescences, and fresh weight of anthers of six plants (*n* = 6) per each condition were recorded. Plant height of each condition was recorded at 0 and day 7. Number of anthers were randomly counted from four plants (*n* = 4) per each condition. Pollen-containing anthers were manually removed from inflorescences undergoing anthesis using 75% (v/v) ethanol-sterilized forceps, and placed in 1.5 ml microcentrifuge tubes. The samples were stored at −80°C for further analyses.

Pollen grain counting, pollen morphology study, and pollen size measurement were performed on fresh pollen on the day of pollen collection. One milliliter of Calberla's solution as mounting media was added to stain pollen grains. The sample was placed on a glass slide and the microscopic observations were performed using the Olympus BX43 (Olympus Corp., PA, USA) compound microscope attached with the Olympus DP11 digital camera (Olympus Corp., PA, USA) at the magnification of 40x. To count pollen grains, an individual anther from four selected anther (*n* = 4) per each treatment was observed. Pollen morphology and pollen size were studied in rice only for the photographs. Length and width of 20 rice pollen grains (*n* = 20) per each sample group was measured using Image J (NIH Image, MD, USA).

### Total RNA Extraction and cDNA Synthesis

Total RNA was extracted using TRIzol^TM^ Reagent kit (Invitrogen, CA, USA). About 50–100 mg of pollen-containing anthers were rapidly ground into a fine powder using a mortar and pestle that was pre-cooled with liquid nitrogen. The total RNA was purified with FavorPrep^TM^ After Tri-Reagent RNA Clean-Up Kit (Favorgen Biotech Corporation, Ping-Tung, Taiwan). The purified RNA was used as a template for first-strand cDNA synthesis using iScript^TM^ cDNA Synthesis Kit (Bio-Rad, CA, USA) according to manufacturer's instructions. The cDNA was synthesized from 500 ng RNA using oligo-dT primer and iScript reverse transcriptase (Bio-Rad, CA, USA). Concentration and purity of the cDNA were assessed by NanoDrop 2,000 Spectro-photometer (Thermo Scientific, MA, USA). The cDNA was stored at −20°C until used.

### Quantitative Real-Time RT-PCR

Quantitative RT-PCR (qRT-PCR) was used to quantify the amount of specific cDNA in each sample. The concentration of cDNA template was adjusted to 650 ng/ml. All specific primers for β-expansin and reference genes: actin (*ACT1* for rice and *ACT2* for maize) and ubiquitin (*UBQ10* for rice and *UBQ7* for maize), used in this study are listed in Table 1. Each PCR reaction was mixed thoroughly and then added into the MicroAmp^TM^ Optical 96-Well Reaction Plate (Applied Biosystems, CA, USA). Samples were analyzed in triplicates using 7,500/7,500 Fast Real-Time PCR Systems (Applied Biosystems, CA, USA).

**Table 1:** Primer sequences for either β-expansin or reference genes of rice and maize.

**Gene**	**Primer sequence (5**^**′**^**—>** **3**^**′**^**)**		**References**
	**Forward**	**Reverse**	
*OsEXPB*	AAGGATGGCAAGGACGAAGA	CATCACCAGCGACGTACTTG	This study
*OsACT1*	CTGCGGGTATCCATGAGACT	TGGAATGTGCTGAGAGATGC	(41)
*OsUBQ10*	TGGTCAGTAATCAGCCAGTTTGG	GCACCACAAATACTTGACGAACAG	(42)
*ZmEXPB*	ACGAGGTGAGATGCAAGGAA	GAACTCGACGTCCATGATGC	This study
*ZmACT2*	CTGAGGTTCTATTCCAGCCATCC	CCACCACTGAGGACAACATTACC	(43)
*ZmUBQ7*	CAGACTACAACATCCAGAAG	TATTAGACGACGACATCCATA	(44)

*Os, Oryza sativa (rice); Zm, Zea mays (maize); EXPB, β-expansin; ACT1, actin-1; UBQ10, ubiquitin-10; ACT2, actin-2; UBQ7, ubiquitin-7*.

### Rice Pollen Crude Protein Extraction

Before pollen protein was extracted, the collected pollen-containing anthers of six plants from each treatment condition were pooled together and then split into three replicates (*n* = 3). Sample pooling was necessary because the amount of pollen obtain from each plant was limited, and variable. Despite of the fact that the experiment was performed with pooled pollen samples from six plants for each stress condition, each pollen sample was obtained from more than a hundred anthers derived from several rice culms that developed independently, each anther was considered as independent sample. Approximately 25 mg of −80°C frozen rice pollen (within 120–150 anthers) was put into a mortar and ground rapidly at room temperature. Two hundred fifty microliters of extraction buffer [1X phosphate-buffered saline (PBS), pH 7.0 at 4°C], was added. The pollen in the extraction buffer was continuously ground to extract the pollen proteins. The duration of extraction was totally in 10 min. To stop protease enzymatic reactions, ten microliters of 1 mM PMSF was added into the sample. Crude pollen extract was centrifuged at 10,000 rpm for 10 min at 4°C to remove insoluble particles. The supernatant was collected into a new microcentrifuge tube. Soluble protein concentration was determined by Bradford assay in comparison with Bovine Serum Albumin (BSA) (Sigma-Aldrich, MO, USA) standard curve. Light absorbance was detected at 595 nm by Biochrom EZ Read 400 Microplate Reader (Biochrom, Cambridge, UK) and quantified by Galapagos Data Acquisition Software (Harvard Bioscience, Inc., MA, USA). The evaluated OD was measured in three replications.

### Sodium Dodecyl Sulfate-Polyacrylamide Gel Electrophoresis With Coomassie Blue Staining

Crude pollen protein extract of experimental rice in 1X PBS was incubated at 95°C for 5 min before being separated using 14% separating and 7% stacking gel electrophoresis. Ten micrograms of the rice protein extract were loaded into each well. For visualization, the SDS-PAGE gels were stained with 0.3% (w/v) Coomassie Brilliant Blue R-250 (Merck, NJ, USA) solution and destained with destaining solution [40% (v/v) methanol and 10% (v/v) acetic acid].

### Serum Samples and Ethics Approval

Serum samples were obtained from a collection of 126 donors: 104 patients diagnosed with allergic rhinitis (AR) and 22 healthy donors, with the informed consent as a part of the project number 758/2559 (EC2), in accordance with the approved ethics for research in humans by the Human Research Ethic Committee of Siriraj Hospital, Mahidol University, Bangkok, Thailand. Grass pollen sensitization was assessed based on clinical diagnosis by skin-prick test (SPT) with three common allergenic grass pollen extracts: Bermuda (*Cynodon dactylon*), Johnson (*Sorghum halepense*) and Para (*Urochloa mutica*) grasses performed at the ENT Allergy Clinic, Siriraj Hospital.

Of all 104 AR patients, 80 subjects were sensitized to at least one grass pollen extract with the wheal size ≥3 × 3 mm were considered to have positive test results. All healthy donors showed negative skin test to all three grass pollen extracts (wheal size <3 × 3 mm) were considered as negative controls. Serum samples were collected from each participant, aliquoted, and stored at −20°C until use.

Among all sensitized subjects and negative controls, eight patients and negative controls gave consent for further research studies. Four patient sera with highest IgE reactivity to at least one grass pollen extract and two control sera with no IgE reactivity to all grass extracts, and enough serum volume available were chosen for this study.

### Immunoblot Assay

One SDS-PAGE gel with four rice pollen extract samples: untreated control, flood, salt, and drought, was prepared for each serum sample. Ten micrograms of the pollen extract quantified by the Bradford assay were separated by SDS-PAGE as described above. The separated proteins were electro-transferred from gels to nitrocellulose membranes using Trans-blot® Turbo^TM^ Transfer System (Bio-Rad, CA, USA) with the manufacturer's protocol. The membranes were blocked with 3% (w/v) Skim Milk Powder (Merck, NJ, USA) in 0.2% (v/v) phosphate buffered saline with Tween® 20 (Sigma-Aldrich, MO, USA) (PBST) as a blocking buffer at room temperature for 1 h. Each membrane was incubated with individual serum sample (primary antibody) diluted in a blocking buffer (1:20), shaken gently at 4°C overnight. The individual membrane was then incubated at room temperature for 1 h with 1 ml of HRP-labeled mouse IgG anti-human IgE antibodies (SeraCare Life Sciences, MA, USA) (secondary antibody) diluted in blocking buffer (1:5,000). Bound IgEs were detected using Immobilon^TM^ Western Chemiluminescent HRP substrate (Millipore, Germany) and visualized by a gel documentation system (ImageQuant LAS 500, GE Healthcare Life Sciences, MA, USA) with 1 min of exposure time. The molecular weight of the reactive protein bands was estimated by comparison with the standard protein molecular weight markers, BLUeye Prestained Protein Ladder (GeneDireX, Keelung, Taiwan), on the developed SDS-PAGE gels. The immunoblot assay was performed once due to serum limitation.

### Indirect Enzyme-Linked Immunosorbent Assay

Each experimental rice pollen extract was diluted with 1X PBS to a concentration of 1 μg per well. One hundred microliters of the diluted protein sample was coated on ELISA plates and incubated at 4°C overnight. The coated plates were washed five times with a washing buffer (0.05%, v/v, Tween in 1X PBS) and blocked with a blocking solution (1%, w/v, skim milk powder in 0.05% PBST) for 1 h at room temperature. The coated plates were then incubated with diluted serum (1:4) of either allergic donors (*n* = 4) or non-allergic donors (*n* = 2) for 2 h at room temperature. The bound IgEs were detected by incubating with 1:1,000 diluted HRP-labeled mouse IgG anti-human IgE antibodies in blocking solution at room temperature for 1 h. Fifty microliters of TMB (3,3′5,5-tetramethylbenzidine) substrate (SeraCare Life Sciences, MA, USA) was added to each well and incubated in dark condition at room temperature for 30 min. The enzymatic reaction developed a blue color with increasing IgE interaction and was stopped by 1 N HCl. Light absorbance was measured at optical density (OD) of 450 nm using Biochrom EZ Read 400 Microplate Reader (Biochrom, Cambridge, UK) with Galapagos Data Acquisition Software (Harvard Bioscience, Inc., MA, USA). The OD levels were obtained by subtracting the sample OD with its negative control (no allergen). Each of the three extracts was then tested with each serum sample in three separate reactions, for the total of nine reactions per serum sample. Each data point on the graph was the average value from nine OD readings.

### Statistical Analysis

All statistical analyses were conducted by GraphPad Prism version 9.0.0 (GraphPad Software, CA, USA). The assumptions of normality and homogeneity of variance were tested using Shapiro-Wilk test and Levene's test, respectively. The statistical analysis of physiological characteristics for each treatment group was tested by one-way analysis of variance (ANOVA) with Tukey's multiple-comparison test. The comparison of plant heights between 0 and day 7 was tested by paired sample *t*-test. The significant difference of expression of β-expansin relative to the reference genes, *ACT* and *UBQ*, of experimental rice and maize was analyzed using Kruskal-Wallis test with Dunn's multiple-comparison test. The statistical significance of soluble pollen protein concentration for each experimental rice group was tested by one-way ANOVA with Fisher's least significant difference (LSD) multiple-comparison test.

Demographic data: age, gender, smoking status, pet, family history of allergic disease, and current medication was presented as either mean ± standard deviation (SD) and range for continuous data or frequency and percentage for categorical data. The demographic data of grass-allergic subjects and healthy control subjects was compared using the student's unpaired *t*-test (for continuous data) or Fisher's exact test (for categorical data).

The IgE levels of four grass-allergic patients (*n* = 4) and two healthy controls (*n* = 2) evaluated by immunoblot and ELISA were analyzed. The significant difference of the immunoblot IgE-reactive band intensity and ELISA OD between patients and controls was analyzed for each stress condition using Mann-Whitney *U*-test (median used for the analysis of non-parametric data). To compare IgE reactivity levels between rice pollen extracts in each stress condition for each subject group, one-way ANOVA was used with Tukey's multiple-comparison test for grass-allergic patients (average values used for the analysis of parametric data) and Kruskal-Wallis *H*-test with Dunn's multiple-comparison test was used for healthy control donors (average rank used for the analysis of non-parametric data).

A *p*-value of <0.05 (*p* < 0.05) was considered statistically significant.

## Results

**Figure 2:**
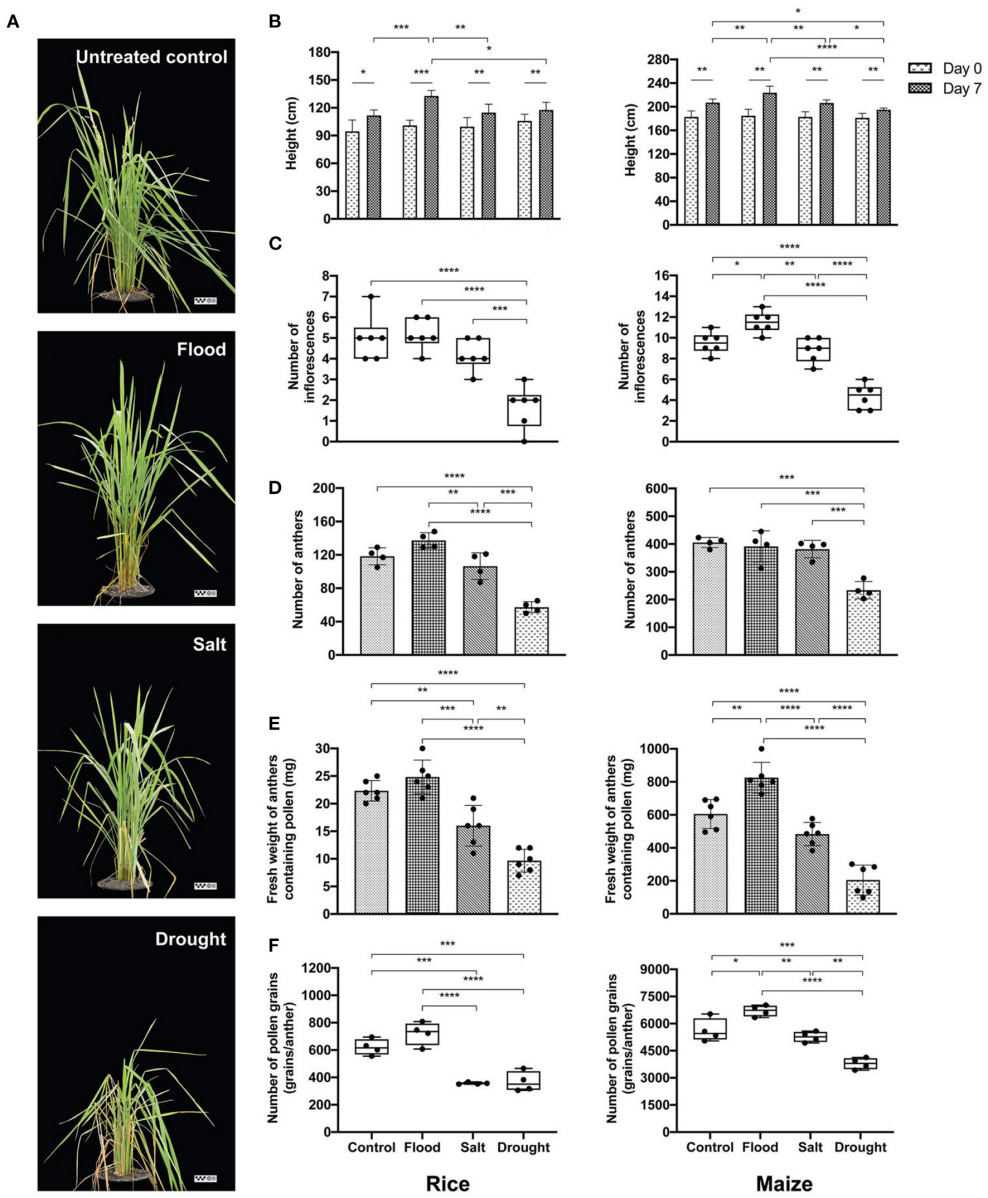
Effects of abiotic stress on experimental plants. Morphological characteristics of untreated control, flood-, salt-, and drought-treated rice were observed at day 7 (the reference scale: 25 cm) **(A)**. Physiological variations of experimental rice (left panel) and maize (right panel) were recorded as follows: plant height at 0 and day 7 **(B)**, number of inflorescences **(C)**, number of anthers **(D)**, weight of anthers containing pollen **(E)**, and number of pollen grains **(F)**. Bars with error bars represent mean and SD. Box plots with error bars represent median and interquartile range. Comparing plant heights between 0 and day 7 of each treatment was tested by paired sample *t*-test. Comparing other characteristics between control, flood, salt, and drought was tested by one-way analysis of variance (ANOVA) (parametric test) with Tukey's multiple-comparison test. A statistically significant difference was indicated with the following asterisks: *(*p* < 0.05), **(*p* < 0.005), ***(*p* < 0.001), and ****(*p* < 0.0001).

Rice and maize plants given flood, salt, or drought stress for 1 week were investigated to confirm the extent of physiological changes caused by the stresses. Overall, the stress treatments resulted in visible symptoms such as dried leaves in rice, particularly under drought conditions (Figure 2A). On the contrary, inflorescence and pollen shapes did not change significantly compared with the untreated control (Supplementary Figure 1). Interestingly, although the rice pollen morphology under all treatment conditions was comparable, the pollen size affected by salt treatment was significantly larger compared to that of the drought treatment (width: *p* < 0.05; length: *p* < 0.0001) (Table 2).

**Table 2:** Pollen size of rice under each experimental condition.

**Condition**	**Pollen size (μm)** ***^†^,^††^,^†††^***	
	**Width**	**Length**
Control	40.4 ± 3.1^a^	43.8 ± 2.3^a^
Flood	40.3 ± 2.8^a^	43.4 ± 2.4^a^
Salt	41.3 ± 2.9^b^	45.7 ± 3.6^b^
Drought	38.2 ± 3.3^a^	41.6 ± 2.5^a^

† *Measured from 20 grains per sample group*;

†† *Mean ± SD*;

††† *One-way ANOVA with Tukey's multiple-comparison test was used to compare either pollen width or length of each experimental group*.;

a, b *Different letters correspond to statistically significant differences at 95% C.I*.

Plant height, number of inflorescences, number of anthers, fresh weight of anthers, and number of pollen grains were recorded from stress-treated rice and maize plants (Figures 2B–F). At the day of treatment, rice and maize plants had uniform height (*p* > 0.05). After 1 week of treatments, the maximum height was observed under flood condition in both rice and maize (Figure 2B). Drought treatment reduced maize plant height more than control (*p* < 0.05), flood (*p* < 0.0001), and salt (*p* < 0.05) (Figure 2B, right panel**)**.

The number of inflorescences in flood-treated and salt-treated rice was comparable with that in untreated control with an average of five inflorescences/plant, while the drought-treated rice plants had the lowest number of inflorescences (average: two inflorescences/plant) (Figure 2C, left panel). Each untreated- and salt-treated maize plant produced about nine inflorescences. The inflorescence number was highest in flood-treated maize plants and lowest in drought-treated maize plants (Figure 2C, right panel).

Similarly, anther number was significantly affected by stress treatments. More anthers were produced in rice under flooding stress than under salt stress and untreated control. The numbers of maize anthers were comparable in the untreated, flood-treated, and salt-treated groups. However, lower quantities of both rice and maize anthers were recorded in the drought-treated group (Figure 2D). The fresh weight of rice anthers was the highest in flood-treated samples at about 25 mg/plant. On the other hand, the weight of drought-treated anthers, which were the lightest of all samples, was 60% less than the weight of flood-treated anthers. The maximum weight of maize anthers was also produced by maize under flood stress (800 mg/plant), which was about 4-fold higher than the anther weight in the drought-treated group (Figure 2E). When the number of pollen grains per anther was counted, it was found that rice plants under salt and drought treatments produced an average of 400 grains/anther. This level was about half the amount of pollen produced by flood-treated rice plants (Figure 2F). The largest quantity of pollen grains was recorded from flood-treated maize plants (average: 6,700 grains/anther).

**Figure 3:**
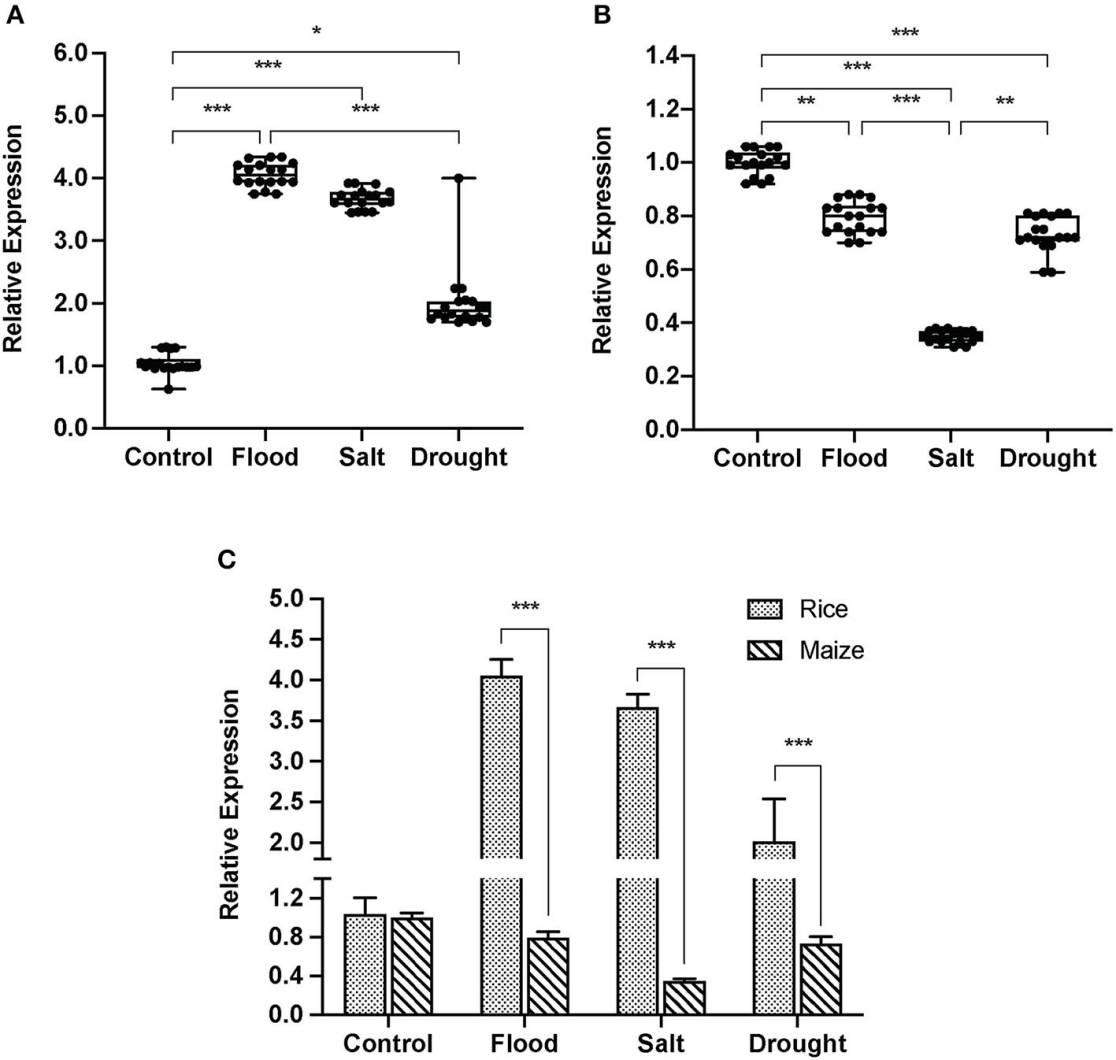
Relative expression of group-1 expansin, β-expansin in the experimental rice and maize pollen. The expression was relative to the reference genes: *ACT* (*ACT1* for rice and *ACT2* for maize) and *UBQ* (*UBQ10* for rice and *UBQ7* for maize). The box plot showed the relative expression of the experimental rice **(A)** and maize **(B)** under either untreated, flood-, salt-, or drought-treated conditions. Box plots with error bars represent median and interquartile range. The comparative diagram **(C)** showed the mean relative expression of β-expansin in the experimental rice and maize. Bars with error bars represent mean and SD. A statistically significant difference tested by Kruskal-Wallis test with Dunn's multiple-comparison test was indicated with the following asterisks: *(*p* < 0.05), **(*p* < 0.005), and ***(*p* < 0.0001).

Expression of reference genes, *ACT* (*ACT1* for rice and *ACT2* for maize) and *UBQ* (*UBQ10* for rice and *UBQ7* for maize), remained stable in both rice and maize among the four experimental conditions (no statistically significant difference between the treated conditions; rice: *p* = 0.26; maize: *p* = 0.41; data not shown). Relative expression of β-expansin significantly increased in pollen of all stress-treated rice (Figure 3A). The highest level of β-expansin transcript (4-fold increase compared to the untreated control) was found in flood-treated rice pollen (*p* < 0.0001). Drought treatment increased β-expansin expression in rice pollen ~2-fold (*p* < 0.05), whereas salt treatment increased the expression 3.7-fold (*p* < 0.0001) compared to the control. On the contrary, all stress treatments decreased the expression levels of β-expansin in maize pollen by the average of 20% (Figure 3B). The minimum transcript level of β-expansin was found under salt stress conditions and was nearly 60% lower than the transcript level in untreated maize pollen.

When the basal expression level of β-expansin in rice and maize was adjusted to one unit (Figure 3C), five times higher expression level was found in flood-treated rice compared to the flood-treated maize. Under salt and drought conditions, the β-expansin expression levels in rice pollen were ~3-fold higher than those in maize pollen.

Immunoblotting and indirect ELISA were performed to determine whether the increase of RNA expression in the rice pollen after stress treatments was consequently reflected by the increase of grass-group 1 allergenic proteins, and thereby could increase the IgE reactivity of the pollen. Serum samples of four grass-allergic patients and two healthy control donors, with demographic data summarized in Table 3 and Supplementary Table 1, were used for immunoblotting and ELISA. The majority of patients and control donors were adults (average age of patients: 34.5 ± 13.9; control donors: 30.5 ± 9.19 years). All patients took antihistamine and/or intranasal corticosteroid as prescribed. Average age, gender distribution, smoking and pet status, family history of allergic diseases, and medication were not different between patients and control donors.

**Table 3:** Demographic data of patient and control subjects recruited in this study.

	**Patients** **(*n* = 4)**	**Controls** **(*n* = 2)**	***P*-value** **^†^**
**Age (years)**			
Mean ± SD	34.5 ± 13.9	30.5 ± 9.19	0.738 (NS)
**Gender**			
Male (%)	0 (0%)	1 (50%)	0.178 (NS)
Female (%)	4 (100%)	1 (50%)	
Smoking (%)	2 (50%)	0 (0%)	0.467 (NS)
Pet (%)	2 (50%)	0 (0%)	0.467 (NS)
Family history of allergic disease (%)	2 (50%)	0 (0%)	0.467 (NS)
Medication^††^ (%)	4 (100%)	0 (0%)	0.067 (NS)

† *To compare the demographic data between patients and controls, student's t-test was used for age and Fisher's exact test was used for gender, smoking, pet, family history of allergic disease, and medication.; NS, non-statistical difference at 95% C.I*.;

†† *Current medication with antihistamine and/or intranasal corticosteroid*.

**Figure 4:**
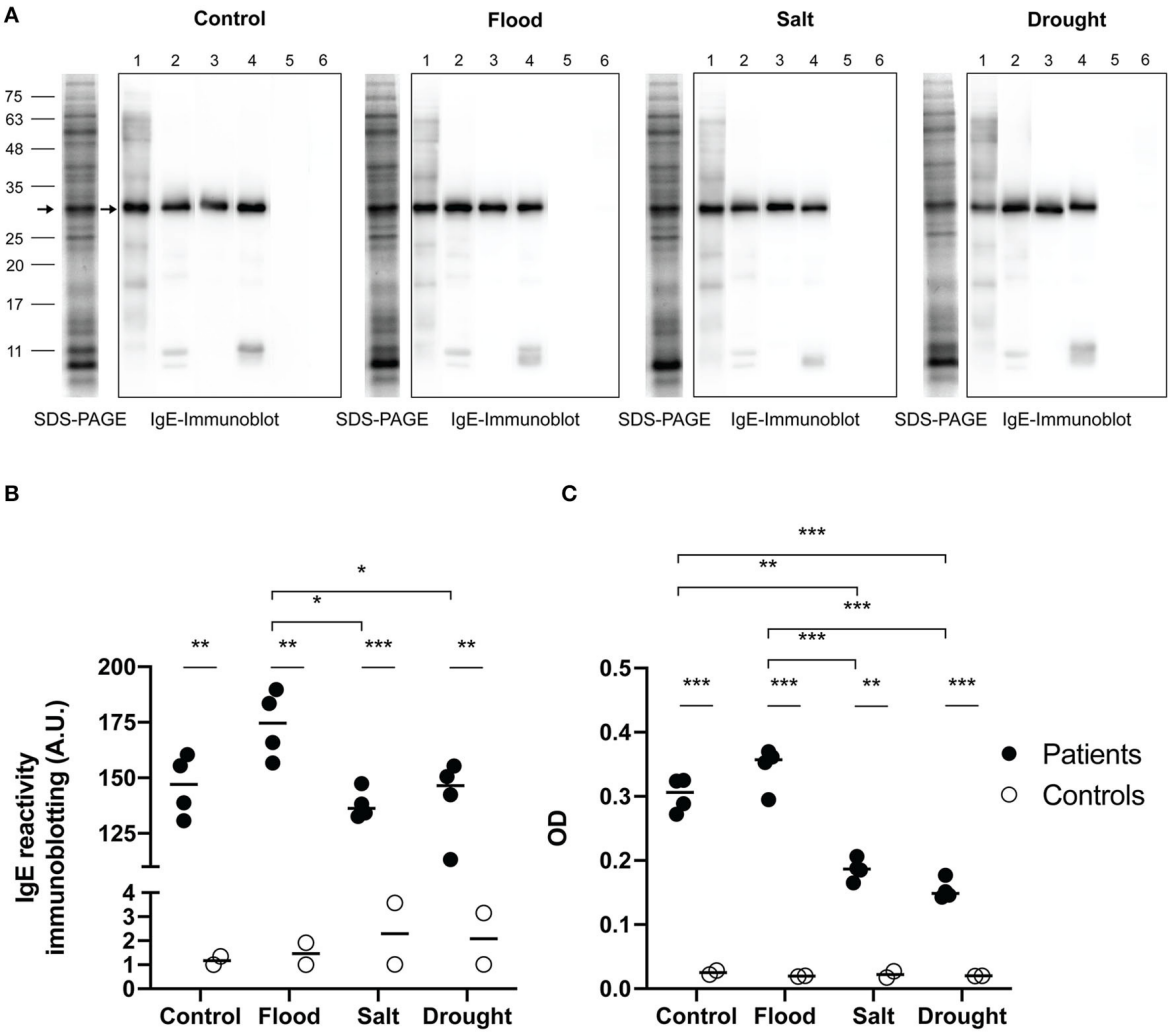
IgE reactivity to rice pollen protein extracts. SDS-PAGE and IgE immunoblots **(A)** of rice pollen protein extracts under stress conditions from four representative patient samples for grass pollen (no. 1–4) and two representative control samples (no. 5–6), were shown. Arrows on IgE immunoblots indicate major IgE reactive bands. An arrow on each SDS-PAGE indicated the corresponding protein band. The interleaved scatter plot showed intensity of IgE immunoblot **(B)** and ELISA OD **(C)** indicated IgE reactivity between grass-allergic patients (*n* = 4) and controls (*n* = 2) to each of the experimental rice pollen extracts were illustrated. Comparing IgE level between grass-allergic patients and controls to each of the experimental rice pollen extracts was tested by Mann-Whitney *U*-test. Comparing IgE level between control, flood, salt, and drought rice pollen extracts was performed by one-way ANOVA with Tukey's multiple-comparison test for parametric data or Kruskal-Wallis *H*-test with Dunn's multiple-comparison test for nonparametric data. A statistically significant difference was indicated with the following asterisks: *(*p* < 0.05), **(*p* < 0.001), and ***(*p* < 0.0001).

The maximum soluble protein concentration of rice pollen extract assessed by Bradford assay was found in the salt-treated samples followed by drought, flood, and control samples (Table 4). IgE-immunoblotting was used to study the profile of IgE reactivity to grass group-1 pollen allergenic proteins. The most prominent IgE-reactive protein bands of rice pollen under flood, salt, drought and control treatments were ~30 kDa (4/4, 100% of patients). Two minor bands at 11 kDa and slightly lower were also detected in 2/4 patient serum samples (Figure 4A). In all treatments, there was no detectable IgE reactivity of sera from healthy control donors to rice pollen extract. Consequently, IgE reactivity to the rice pollen extract of sera from grass-allergic subjects were different from those from control donors in both immunoblotting assay and ELISA (Figures 4B,C).

**Table 4:** Soluble protein concentration of rice pollen extract.

**Condition**	**Concentration (mg/ml)^†^, ^††^**
Control	4.19 ± 0.64^a^
Flood	4.26 ± 0.48^a^
Salt	5.66 ± 0.74^b^
Drought	5.48 ± 0.65^b^

† *Mean ± SD*;

†† *One-way ANOVA with Fisher's least significant difference (LSD) multiple-comparison test was used to compare soluble protein concentration of each experimental group*.;

a, b *Different letters correspond to statistically significant differences at 95% C.I*.

The intensity of IgE reactivity to the 30 kDa protein among the grass-allergic patient serum samples, corresponding to the grass group 1 major allergen/β-expansin, was significantly higher (*p* < 0.05) in extracts from rice pollen subjected to flood treatment than those from salt-treated or drought-treated rice pollen (Figure 4B). A trend was observed that the flood treatment slightly increased the IgE reactivity compared to the untreated control, although the difference was not statistically significant (*p* > 0.25).

Indirect ELISA was used to assess IgE levels in sera of grass-allergic subjects against rice pollen extracts (Figure 4C). The IgE level of allergic subjects against salt rice pollen extract or drought rice pollen extract showed a significant decrease compared to the level of IgE against control rice pollen extract. The allergic subjects have a significantly higher level of IgE to flood rice pollen extract compared to either salt or drought rice pollen extracts. Both immunoblotting and ELISA assays produced similar results, and generally corresponded with the β-expansin expression results with the exception of the low basal expression in the untreated pollen samples.

## Discussion

Since the beginning of the 21st century, the incidence of grass pollen sensitization worldwide, particularly in Thailand has been rising continuously (45). Importantly, grass pollen allergens, particularly the group-1/β-expansins, are reported as a major cross-reactive protein for allergic patients (14). Apart from the amounts of pollen released into the air, allergenic potency of pollen can also influence the prevalence and/or severity of allergic diseases (15). In addition, anthropogenic and environmental factors can lead to changes of plant distribution, plant adaptation, and the volumes of pollen released into the atmospheric air.

This study demonstrates the effects of environmental stresses on plant physiological characteristics, β-expansins expression, and IgE reactivity to major rice pollen allergens. The visible changes in plant growth, development, and productivity, including plant height, as well as number of anthers and pollen grains resulting from either flood, salt, or drought stresses is consistent with previous studies (46–48).

The changes in physiological characteristics of rice and maize were indicative of mild-to-moderate stress as intended by the stress treatments (49, 50). Importantly, these stresses are comparable to what the plants are exposed to occasionally during the pollen-forming stages and are likely to experience more frequently as the result of climate change in the near future (51). These physiological changes can also serve as a reference for further research investigating other cultivated or naturally distributed grass species.

The expression of β-expansin transcript in the experimental rice pollen under flooding, salt, and drought conditions was significantly induced compared to the control pollen not subjected to stress, whereas the expression in all pollen samples from stress-treated maize was obviously repressed relative to the control.

For rice, the highest upregulation level of β-expansin was observed in the flood-treated group followed by salt- and drought-treated groups, respectively. According to several previous observations, the increase in expression level of expansin genes, particularly β-expansin in stress-treated plants were shown to correlate with the maintenance of plant growth or stress relaxation, in accordance with the function of β-expansin in plant cell-wall modification (28, 52–54).

However, the expression patterns of β-expansin in all stressed maize tended to be reduced. The maximum level of β-expansin transcript was detected in the untreated control samples, whereas the lowest expression level was exhibited in the salt-treated samples. Corresponding to the previous study (55), the relative expression levels of several β-expansin isoforms, *ZmEXPB2, ZmEXPB6, ZmEXPB7*, and *ZmEXPB8*, in 100 mM NaCl-treated maize were significantly lower compared to the expression levels in 1 mM-NaCl-treated control. Moreover, the Western blot analysis revealed a decrease of β-expansin protein detected in response to the 100 mM NaCl treatment (55). Based on our findings, it was hypothesized that the downregulation of β-expansin transcripts may contribute to the decreased abundance of β-expansin protein accompanying shoot growth defects during high-salt treatment (55, 56).

Notably, several stress-responsive genes encode pollen allergens. For instance, several isoforms of Bet v 1, a major allergen in birch (*Betula pendula*) pollen, are encoded by multiple *PR10* genes such as *Bet v 1* genes belonging to the pathogenesis-related gene family that play a role in biotic and abiotic stress responses (57). β-expansins encoded by β-expansin genes also belong to major grass pollen allergens that impact on a large number of grass allergic individuals worldwide. This pollen allergen as well as its allergenicity were speculated to be influenced by either up- or down-regulation of plant β-expansin transcripts under unfavorable environmental conditions.

The comparison of β-expansin expression patterns across the two plant species: rice and maize, revealed highly different transcriptional dynamics in response to either flood, salinity, or drought. This is in agreement with several previous investigations indicating that the expression of different sets of genes can be variable across plant species in response to various environmental or stress conditions (58, 59). For instance, salt-responsive genes in rice and barley showed variations in types or levels of expression during salt treatment (60).

The profiles of IgE-immunoblotting emphasized that IgE serum samples of all grass allergic patients were reactive to the β-expansin-like protein indicated at a molecular weight of about 30 kDa in all experimental rice pollen extracts. The immunoblot results showed that the stress treatments affected the levels of β-expansin-like protein, but did not noticeably lead to increased production of other IgE-reactive proteins. The relative band intensity of patient IgE against the flooded rice pollen extract was significantly higher than either salt-treated or drought-treated rice pollen extracts but was comparable to the control. The OD levels assessed from the indirect ELISA showed a similar trend of relative intensity. Based on ELISA, the IgE reactivity against the major rice pollen allergens fluctuated. Higher levels of IgE were observed against either flood-treated or untreated rice pollen extracts whereas lower levels of the IgE were detected against either salt-treated or drought-treated rice pollen extracts. Generally, ELISA is quantitative and more favorable for certain purposes, but the semi-quantitative IgE-immunoblotting also has advantages in that the proteins are separated by size, and therefore reactivity to specific protein bands can be selectively assessed without the confounding background.

Although grasses are dominant allergenic species in most regions of the world, most allergenic grass species distributed naturally are not easily cultivated in a greenhouse for pollen collection and gene expression analyses. Moreover, several species and/or cultivars cannot be verified due to their high taxonomic diversity. Therefore, in this study of immunoreactivity to grass pollen allergens, rice and maize were chosen due to their certified genetic background, ease of cultivation, and previous literature about gene and protein sequences. In Thailand, like most of Asia, rice is the most widely cultivated grass economic crop species, averaging 11 million hectares in cultivation area, which is ~ten times larger than maize cultivation area (1.1 million hectares) (61). Therefore, most people throughout the country, especially occupational workers such as farmers can be highly exposed to large amount of rice pollen. Due to the limitation of serum samples in this study, rice was, therefore, the only species selected for the analysis of the IgE reactivity immunoblot assay.

The study of rice pollen immunoreactivity has still been limited and rice pollen is likely well-tolerated by the immune system of local farmers. However, the grass group-1 allergens have high potential to cause severe allergenicity in grass pollen-allergic patients and high frequency (>90%) of patients reacted to them. Group-1 grass allergens from pollen of different subtropical grasses frequently found in Southeast Asia were shown to be nearly identical (97.79–100% identity) (62). Another previous study from Dhammachat et al. (63) showed the high percentage (81%) of sequence identity between grass group-1 allergen in rice (Ory s 1) and in Para grass (Uro m 1.03), another dominant allergenic grass species found in Thailand. Because of the large cultivation area of rice and maize and the highly similar allergenic protein sequence among tropical/subtropical grass species, the fluctuation of rice and maize pollen is likely to have clinical relevance for patients already sensitized to grass pollen allergens.

The IgE reactivity evaluated by IgE immunoblotting and ELISA showed generally consistent results with the relative expression of β-expansin transcripts, with the exception of the basal expression in the untreated control samples. This discrepancy may be associated with the specificity of target samples. For the transcript analysis, the designed primers specific for β-expansin transcript were used to specifically quantify the expression level of β-expansin in pollen samples. However, for the IgE reactivity study, the soluble pollen extract proteins were applied for immunoblotting and ELISA. We hypothesized that the rice pollen extract containing several protein isoforms of β-expansin as well as other minor allergens could react to the patient IgE, resulting in high background readings in control samples.

Apart from the influence of other β-expansin isoforms interacting with patient IgE, the effects of various stress-related proteins being expressed under stress conditions are worth a discussion. Due to the evaluation of IgE reactivity to the experimental rice pollen extracts using the soluble pollen extract proteins, the highest protein concentration (5.66 mg/ml) was found in the salt-treated group followed by drought (5.48 mg/ml), and flood (4.26 mg/ml) treated groups, as well as the untreated-control group (4.19 mg/ml), respectively. According to the results of IgE reactivity, the immunoblotting and ELISA of salt- and drought-treated samples showed a significant low reactivity in spite of their high soluble protein concentrations. Those observations were different from the IgE reactivity of flood-treated and untreated control groups. The results suggested that the soluble protein concentrations were not agreeable with the Ig reactivities. We hypothesized that the high amount of soluble protein concentrations could be affected by an increase of large amounts of other stress-related proteins, including heat-shock and dehydration responsive-element binding (DREB) proteins, in response to abiotic stresses (64–66). According to several previous studies, for example, the overexpression of *OsDREB* genes and also non-allergenic OsDREB proteins was found in most parts of rice, particularly in spikelets and anthers under drought stress (67–70). This result could reduce the proportion of β-expansin in the experimental samples, particularly salt- and drought-sample groups contributing to a decrease in IgE reactivities.

The amount of major pollen allergen could fluctuate among plant species and environmental conditions, including climatic variations and/or atmospheric pollution (15, 32, 71). Our findings revealed similar evidence compared to several previous studies. For instance, an increase of Amb a 1 transcript was observed in ragweed pollen exposed to a high level of CO_2_ and treated with drought in accordance with the Amb a 1 protein content in ragweed pollen that was also found to increase under the stress conditions (32, 72).

Another interesting study of environmental effects on plant allergens showed that a Phl p 5 protein in timothy grass pollen could be altered by oxidative damage resulting from high O_3_ exposure. This factor contributed to a reduction in the human IgE antibody recognition as observed from the 2D-gel allergen pattern of O_3_-exposed pollen (73, 74). Under a drought stress condition, several previous investigations indicated that, for example, rice treated with either water deficit or salinity (120 mM of NaCl) for at least 8 days had significant increases in superoxide anion, hydrogen peroxide, and malondialdehyde (a highly reactive compound acting as a marker for oxidative stress) (75–80). In keeping with previous literature, an increase in oxidative damage in stress-treated plants causes several protein-conformation changes and/or denaturation (81, 82). Our findings, particularly in drought- and salt-stress-treated rice, indicated a lower level of IgE reactivity compared to flood-treated and untreated-control rice. It is likely that the β-expansin conformation in salt- or drought-treated conditions could be altered and/or damaged by oxidative stress at the equivalent concentration of loaded proteins. In effect, this may contribute to a decrease in the allergen recognition by IgE in addition to the reduction of detectable IgE reactivity to the stress-treated rice β-expansin.

Another study of hypersensitivity reactions in allergic conjunctivitis influenced by ragweed pollen-induced oxidative stress revealed a different aspect. Experimental mice challenged with ragweed pollen pretreated with a superoxide scavenger Tiron showed a significant decrease in mast cell degranulation compared to the ragweed pollen treated with NAD (P) H oxidases. The authors presumed that the stimulation of mast cell degranulation in the conjunctiva is significantly induced by ROS from NAD (P) H oxidase treated ragweed pollen together with the pollen allergens (83). Remarkably, several previous studies demonstrated that environmental fluctuations can govern both the expression of gene-encoding major pollen allergens and the allergenicity of pollen, similar to the findings in this study.

## Conclusion

As expected, rice and maize plants subjected to environmental stresses during pollen development, particularly drought stress, exhibited significant differences in plant height, anther fresh weight, number of inflorescences, anthers, and pollen grains. The stress conditions also elicited the changes in β-expansin gene expression. For rice, the β-expansin expression in pollen of flood-treated samples was ~four times higher than that of untreated controls, followed by salt- and drought-treated samples. In contrast, the expression of β-expansin in pollen of maize showed an apparent decrease under all stress treatments. IgE-immunoblot analysis showed IgE-reactive protein bands from rice pollen extracts at about 30 kDa as belonging to the grass-group 1 protein. The intensity of IgE-reactive protein bands and the IgE level to rice pollen extracts showed significant differences among stress conditions, particularly salt and drought stresses compared to the untreated condition. These findings suggest that environmental conditions during plant growth and flowering, which regulate the total β-expansin content, could significantly influence the allergenic potency of rice and maize pollen, and possibly the severity of rice pollinosis. Further studies should be conducted to expand our understanding about effects and consequences of the concerning environmental changes on pollen allergies in the near future.

## Data Availability Statement

The original contributions presented in the study are included in the article/Supplementary Materials, further inquiries can be directed to the corresponding author/s.

## Ethics Statement

The studies involving human participants were reviewed and approved by Human Research Ethic Committee of Siriraj Hospital, Mahidol University, Bangkok, Thailand. The patients/participants provided their written informed consent to participate in this study.

## Author Contributions

WS helped in conceptualizing the research project, supervision, and funding acquisition. YJ and WS designed the study, participated in the data analysis, manuscript writing, original draft preparation, review, editing, and visualization. YJ performed experiments and helped in data curation. All authors contributed to the article and approved the submitted version.

## Conflict of Interest

The authors declare that the research was conducted in the absence of any commercial or financial relationships that could be construed as a potential conflict of interest.

## Publisher's Note

All claims expressed in this article are solely those of the authors and do not necessarily represent those of their affiliated organizations, or those of the publisher, the editors and the reviewers. Any product that may be evaluated in this article, or claim that may be made by its manufacturer, is not guaranteed or endorsed by the publisher.

## References

[1] Bernard SM Samet JM Grambsch A Ebi KL Romieu I. The potential impacts of climate variability and change on air pollution-related health effects in the United States. Environ Health Perspect. (2001) 109:199–209. 10.2307/3435010 11359687 PMC1240667

[2] D'Amato G Liccardi G D'Amato M Holgate S. Environmental risk factors and allergic bronchial asthma. Clin Exp Allergy. (2005) 35:1113–24. 10.1111/j.1365-2222.2005.02328.x 16164436

[3] Hamaoui-Laguel L Vautard R Liu L Solmon F Viovy N Khvorostyanov D. Effects of climate change and seed dispersal on airborne ragweed pollen loads in Europe. Nat Clim Chang. (2015) 5:766–71. 10.1038/nclimate2652

[4] Davies JM. Grass pollen allergens globally: the contribution of subtropical grasses to burden of allergic respiratory diseases. Clin Exp Allergy. (2014) 44:790–801. 10.1111/cea.12317 24684550

[5] Andersson K Lidholm J. Characteristics and immunobiology of grass pollen allergens. Int Arch Allergy Immunol. (2003) 130:87–107. 10.1159/000069013 12673063

[6] Cosgrove DJ Bedinger P Durachko DM. Group I allergens of grass pollen as cell wall-loosening agents. Proc Natl Acad Sci USA. (1997) 94:6559–64. 10.1073/pnas.94.12.6559 9177257 PMC21089

[7] Cottam GP Moran DM Standring R. Physicochemical and immunochemical characterization of allergenic proteins from rye-grass (Lolium perenne) pollen prepared by a rapid and efficient purification method. Biochem J. (1986) 234:305–10. 10.1042/bj2340305 3718469 PMC1146566

[8] Ferreira F Hawranek T Gruber P Wopfner N Mari A. Allergic cross-reactivity: from gene to the clinic. Allergy Eur J Allergy Clin Immunol. (2004) 59:243–67. 10.1046/j.1398-9995.2003.00407.x 14982506

[9] Gangl K Niederberger V Valenta R Nandy A. Marker allergens and panallergens in tree and grass pollen allergy. Allergo J Int. (2015) 24:158–69. 10.1007/s40629-015-0055-3

[10] Wang T Chen Y Tabuchi A Cosgrove DJ Hong M. The target of β-expansin EXPB1 in maize cell walls from binding and solid-state NMR studies. Plant Physiol. (2016) 172:2107–19. 10.1104/pp.16.01311 27729469 PMC5129719

[11] Laffer S Vrtala S Duchêne M van Ree R Kraft D Scheiner O. IgE-binding capacity of recombinant timothy grass (Phleum pratense) pollen allergens. J Allergy Clin Immunol. (1994) 94:88–94. 10.1016/0091-6749(94)90075-2 8027502

[12] Smith PM Avjioglu A Ward LR Simpson RJ Knox RB Singh MB. Isolation and characterization of Group-1 isoallergens from bermuda grass pollen. Int Arch Allergy Immunol. (1994) 104:57–64. 10.1159/000236709 7950406

[13] Tamborini E Faccini S Lidholm J Svensson M Brandazza A Longhi R. Biochemical and immunological characterization of recombinant allergen Lol p 1. Eur J Biochem. (1997) 249:886–94. 10.1111/j.1432-1033.1997.00886.x 9395340

[14] Pacharn P Songnual W Siriwatanakul U Thongngarm T Reamtong O Jirapongsananuruk O. Beta-expansin of bermuda, johnson, and para grass pollens, is a major cross-reactive allergen for Allergic Rhinitis patients in subtropical climate. Asian Pacific J allergy Immunol. (2019) 37:30–5. 10.12932/AP-071117-0191 29549697

[15] Ghiani A Ciappetta S Gentili R Asero R Citterio S. Is ragweed pollen allergenicity governed by environmental conditions during plant growth and flowering? Sci Rep. (2016) 6:1–8. 10.1038/srep30438 27457754 PMC4960655

[16] Beggs PJ. Impacts of climate change on aeroallergens: past and future. Clin Exp Allergy. (2004) 34:1507–13. 10.1111/j.1365-2222.2004.02061.x 15479264

[17] Frei T Gassner E. Climate change and its impact on birch pollen quantities and the start of the pollen season an example from Switzerland for the period 1969-2006. Int J Biometeorol. (2008) 52:667–74. 10.1007/s00484-008-0159-2 18481116

[18] Frenguelli G. Interactions between climatic changes and allergenic plants. Monaldi Arch Chest Dis Pulm Ser. (2002) 57:141–3. 12357846

[19] Kinney PL. Climate change, air quality, and human health. Am J Prev Med. (2008) 35:459–67. 10.1016/j.amepre.2008.08.025 18929972

[20] Poole JA Barnes CS Demain JG Bernstein JA Padukudru MA Sheehan WJ. Impact of weather and climate change with indoor and outdoor air quality in asthma: a work group report of the AAAAI environmental exposure and respiratory health committee. J Allergy Clin Immunol. (2019) 143:1702–10. 10.1016/j.jaci.2019.02.018 30826366 PMC10907958

[21] Schmidt CW. Pollen overload: seasonal allergies in a changing climate. Environ Health Perspect. (2016) 124:70–5. 10.1289/ehp.124-A70 27035881 PMC4829390

[22] Shea KM Truckner RT Weber RW Peden DB. Climate change and allergic disease. J Allergy Clin Immunol. (2008) 122:443–53. 10.1016/j.jaci.2008.06.032 18774380

[23] Zhang Y Bielory L Mi Z Cai T Robock A Georgopoulos P. Allergenic pollen season variations in the past two decades under changing climate in the United States. Glob Chang Biol. (2015) 21:1581–9. 10.1111/gcb.12755 25266307 PMC4356643

[24] Ziska LH Beggs PJ. Anthropogenic climate change and allergen exposure: the role of plant biology. J Allergy Clin Immunol. (2012) 129:27–32. 10.1016/j.jaci.2011.10.032 22104602

[25] Seki M Kamei A Yamaguchi-Shinozaki K Shinozaki K. Molecular responses to drought, salinity and frost: common and different paths for plant protection. Curr Opin Biotechnol. (2003) 14:194–9. 10.1016/S0958-1669(03)00030-2 12732320

[26] Rastogi S Shah S Kumar R Vashisth D Akhtar MQ Kumar A. Ocimum metabolomics in response to abiotic stresses: cold, flood, drought and salinity. PLoS ONE. (2019) 14:1–26. 10.1371/journal.pone.0210903 30726239 PMC6364901

[27] Rizhsky L Liang H Shuman J Shulaev V Davletova S Mittler R. When defense pathways collide. The response of Arabidopsis to a combination of drought and heat stress. Plant Physiol. (2004) 134:1683–96. 10.1104/pp.103.033431 15047901 PMC419842

[28] Cannarozzi G Weichert A Schnell M Ruiz C Bossard S Blösch R. Waterlogging affects plant morphology and the expression of key genes in tef (Eragrostis tef). Plant Direct. (2018) 2:1–22. 10.1002/pld3.56 31245721 PMC6508588

[29] Nanjo Y Skultety L Uváčková L Klubicová K Hajduch M Komatsu S. Mass spectrometry-based analysis of proteomic changes in the root tips of flooded soybean seedlings. J Proteome Res. (2012) 11:372–85. 10.1021/pr200701y 22136409

[30] Jain S Kumar D Jain M Chaudhary P Deswal R Sarin NB. Ectopic overexpression of a salt stress-induced pathogenesis-related class 10 protein (PR10) gene from peanut (Arachis hypogaea L.) affords broad spectrum abiotic stress tolerance in transgenic tobacco. Plant Cell Tissue Organ Cult. (2012) 109:19–31. 10.1007/s11240-011-0069-6

[31] Jain S Srivastava S Sarin NB Kav NNV. Proteomics reveals elevated levels of PR 10 proteins in saline-tolerant peanut (Arachis hypogaea) calli. Plant Physiol Biochem. (2006) 44:253–9. 10.1016/j.plaphy.2006.04.006 16762558

[32] ElKelish A Zhao F Heller W Durner J Winkler JB Behrendt H. Ragweed (Ambrosia artemisiifolia) pollen allergenicity: SuperSAGE transcriptomic analysis upon elevated CO2 and drought stress. BMC Plant Biol. (2014) 14:1–16. 10.1186/1471-2229-14-176 24972689 PMC4084800

[33] Singer BD Ziska LH Frenz DA Gebhard DE Straka JG. Increasing Amb a 1 content in common ragweed (Ambrosia artemisiifolia) pollen as a function of rising atmospheric CO2 concentration. Funct Plant Biol. (2005) 32:667–70. 10.1071/FP05039 32689165

[34] Podolska G Konopka I Dziuba J. Response of grain yield, yield components and allergic protein content of buckwheat to drought stress. Adv Buckwheat Res. (2007) 12:323–8. Available online at: https://citeseerx.ist.psu.edu/viewdoc/download?doi=10.1.1.508.9684&rep=rep1&type=pdf

[35] Pocock KF Hayasaka Y McCarthy MG Waters EJ. Thaumatin-like proteins and chitinases, the haze-forming proteins of wine, accumulate during ripening of grape (Vitis vinifera) berries and drought stress does not affect the final levels per berry at maturity. J Agric Food Chem. (2000) 48:1637–43. 10.1021/jf9905626 10820071

[36] Fu GF Song J Xiong J Li YR Chen HZ Le MK. Changes of oxidative stress and soluble sugar in anthers involve in rice pollen abortion under drought stress. Agric Sci China. (2011) 10:1016–25. 10.1016/S1671-2927(11)60089-8

[37] Beggs PJ Bambrick HJ. Is the global rise of asthma an early impact on anthropogenic climate change? Environ Health Perspect. (2005) 113:915–9. 10.1289/ehp.7724 16079058 PMC1280328

[38] Ariano R Canonica GW Passalacqua G. Possible role of climate changes in variations in pollen seasons and allergic sensitizations during 27 years. Ann Allergy Asthma Immunol. (2010) 104:215–22. 10.1016/j.anai.2009.12.005 20377111

[39] Bunnag C Jareoncharsri P Tantilipikorn P Vichyanond P Pawankar R. Epidemiology and current status of allergic rhinitis and asthma in Thailand-ARIA Asia-Pacific workshop report. Asian Pacific J Allergy Immunol. (2009) 27:79–86. 19548633

[40] Chinratanapisit S Suratannon N Pacharn P Sritipsukho P Vichyanond P. Prevalence and severity of asthma, rhinoconjunctivitis and eczema in children from the Bangkok area: the global asthma network (GAN) phase I. Asian Pacific J Allergy Immunol. (2019) 37:226–31. 10.12932/AP-120618-0336 30447651

[41] Ji Y Tu P Wang K Gao F Yang W Zhu Y. Defining reference genes for quantitative real-time PCR analysis of anther development in rice. Acta Biochim Biophys Sin. (2014) 46:305–12. 10.1093/abbs/gmu002 24492537

[42] Jain M Nijhawan A Tyagi AK Khurana JP. Validation of housekeeping genes as internal control for studying gene expression in rice by quantitative real-time PCR. Biochem Biophys Res Commun. (2006) 345:646–51. 10.1016/j.bbrc.2006.04.140 16690022

[43] Manoli A Sturaro A Trevisan S Quaggiotti S Nonis A. Evaluation of candidate reference genes for qPCR in maize. J Plant Physiol. (2012) 169:807–15. 10.1016/j.jplph.2012.01.019 22459324

[44] Lin Y Zhang C Lan H Gao S Liu H Liu J. Validation of potential reference genes for qPCR in maize across abiotic stresses, hormone treatments, and tissue types. PLoS ONE. (2014) 9:1–11. 10.1371/journal.pone.0095445 24810581 PMC4014480

[45] Oncham S Udomsubpayakul U Laisuan W. Skin prick test reactivity to aeroallergens in adult allergy clinic in Thailand: a 12-year retrospective study. Asia Pac Allergy. (2018) 8:8–15. 10.5415/apallergy.2018.8.e17 29732293 PMC5931924

[46] Juraimi AS Muhammad Saiful AH Begum M Anuar AR Azmi M. Influence of flooding intensity and duration on rice growth and yield. Pertanika J Trop Agric Sci. (2009) 32:195–208. Available online at: http://psasir.upm.edu.my/id/eprint/4688/1/influence_of_flooding_intensity.pdf

[47] Keotshephile K Oarabile M Thebeetsile M Mike MH. Evaluation of maize yield in flood recession farming in the okavango delta, botswana. African J Agric Res. (2015) 10:1874–9. 10.5897/ajar2014.9353

[48] Munné-Bosch S Alegre L. Die and let live: leaf senescence contributes to plant survival under drought stress. Funct Plant Biol. (2004) 31:203–16. 10.1071/FP03236 32688892

[49] Leksungnoen N Uthairatsamee S Na Takuatuathung C. Germination test on native salt tolerant seeds (Buchanania siamensis Miq.) collected from natural saline and non-saline soil. Thai J For. (2016) 35:1–14. Available online at: http://bioff.forest.ku.ac.th/PDF_FILE/DEC_2016/1.pdf

[50] Shahid SA Rahman K ur. Soil salinity development, classification, assessment, management in irrigated agriculture. In: Pessarakli M, editors. Handbook of Plant Crop Stress. Boca Raton, FL: CRC Press. (2011) p. 23–39.

[51] Raza A Razzaq A Mehmood SS Zou X Zhang X Lv Y. Impact of climate change on crops adaptation and strategies to tackle its outcome: a review. Plants. (2019) 8:1–29. 10.3390/plants8020034 30704089 PMC6409995

[52] Lee Y Kende H. Expression of beta-expansins is correlated with internodal elongation in deepwater rice. Plant Physiol. (2001) 127:645–54. 10.1104/pp.010345.ary 11598238 PMC125099

[53] Sabirzhanova IB Sabirzhanov BE Chemeris A V. Veselov DS Kudoyarova GR. Fast changes in expression of expansin gene and leaf extensibility in osmotically stressed maize plants. Plant Physiol Biochem. (2005) 43:419–22. 10.1016/j.plaphy.2005.01.021 15907695

[54] Tabuchi A Li LC Cosgrove DJ. Matrix solubilization and cell wall weakening by β-expansin (group-1 allergen) from maize pollen. Plant J. (2011) 68:546–59. 10.1111/j.1365-313X.2011.04705.x 21749508

[55] Geilfus CM Zörb C Mühling KH. Salt stress differentially affects growth-mediating β-expansins in resistant and sensitive maize (Zea mays L.). Plant Physiol Biochem. (2010) 48:993–8. 10.1016/j.plaphy.2010.09.011 20970350

[56] Geilfus CM Ober D Eichacker LA Mühling KH Zörb C. Down-regulation of ZmEXPB6 (Zea mays β-Expansin 6) protein is correlated with salt-mediated growth reduction in the leaves of Z. mays L. J Biol Chem. (2015) 290:11235–45. 10.1074/jbc.M114.619718 25750129 PMC4416831

[57] Schenk MF Gilissen LJWJ Esselink GD Smulders MJM. Seven different genes encode a diverse mixture of isoforms of Bet v I, the major birch pollen allergen. BMC Genomics. (2006) 7:1–15. 10.1186/1471-2164-7-168 16820045 PMC1552068

[58] Cortijo S Aydin Z Ahnert S Locke JC. Widespread inter-individual gene expression variability in Arabidopsis thaliana. Mol Syst Biol. (2019) 15:1–16. 10.15252/msb.20188591 30679203 PMC6346214

[59] Gasch AP Yu FB Hose J Escalante LE Place M Bacher R. Single-cell RNA-seq reveals intrinsic and extrinsic regulatory heterogeneity in yeast responding to stress. bioRxiv. (2017) 15:1–28. 10.1101/179093 PMC5746276 29240790

[60] Walia H Wilson C Ismail AM Close TJ Cui X. Comparing genomic expression patterns across plant species reveals highly diverged transcriptional dynamics in response to salt stress. BMC Genomics. (2009) 10:1–13. 10.1186/1471-2164-10-398 19706179 PMC2739230

[61] Office of Agricultural Economics. Agricultural Economic Information. Minist Agric Coop. (2021). Available online at: https://www.oae.go.th/view/1/Information/EN-US (accessed November 1, 2021).

[62] Aud-in S Somkid K Songnuan W. Group-1 grass pollen allergens with near-identical sequences identified in species of subtropical grasses commonly found in Southeast Asia. Medicina. (2019) 55:1–10. 10.3390/medicina55050193 31121985 PMC6571983

[63] Dhammachat S Somkid K Piboonpocanun S Reamtong O Pacharn P Bunnag C. Isoforms of group 1 allergens from a tropical/ subtropical para grass (Urochloa mutica) display different levels of igE reactivity and cross-reactivity. Eur Ann Allergy Clin Immunol. (2019) 51:174–85. 10.23822/EurAnnACI.1764-1489.95 30983309

[64] Benešová M Holá D Fischer L Jedelský PL Hnilička F Wilhelmová N. The physiology and proteomics of drought tolerance in maize: early stomatal closure as a cause of lower tolerance to short-term dehydration? PLoS ONE. (2012) 7:1–17. 10.1371/journal.pone.0038017 22719860 PMC3374823

[65] Verslues PE Sharma S. Proline metabolism and its implications for plant-environment interaction. Arab B. (2010) 8:1–23. 10.1199/tab.0140 22303265 PMC3244962

[66] Lata C Prasad M. Role of DREBs in regulation of abiotic stress responses in plants. J Exp Bot. (2011) 62:4731–48. 10.1093/jxb/err210 21737415

[67] Jin Y Yang H Wei Z Ma H Ge X. Rice male development under drought stress: phenotypic changes and stage-dependent transcriptomic reprogramming. Mol Plant. (2013) 6:1630–45. 10.1093/mp/sst067 23604203

[68] Zhang XX Tang YJ Ma Q Bin Yang CY Mu YH Suo HC. OsDREB2A, a rice transcription factor, significantly affects salt tolerance in transgenic soybean. PLoS ONE. (2013) 8:1–7. 10.1371/journal.pone.0083011 24376625 PMC3869746

[69] Chen JQ Meng XP Zhang Y Xia M Wang XP. Over-expression of OsDREB genes lead to enhanced drought tolerance in rice. Biotechnol Lett. (2008) 30:2191–8. 10.1007/s10529-008-9811-5 18779926

[70] Zhao L Hu Y Chong K Wang T. ARAG1, an ABA-responsive DREB gene, plays a role in seed germination and drought tolerance of rice. Ann Bot. (2010) 105:401–9. 10.1093/aob/mcp303 20100696 PMC2826253

[71] Zhao F Elkelish A Durner J Lindermayr C Winkler JB Ruioff F. Common ragweed (Ambrosia artemisiifolia L.): allergenicity and molecular characterization of pollen after plant exposure to elevated NO2. Plant Cell Environ. (2016) 39:147–64. 10.1111/pce.12601 26177592

[72] Choi YJ Oh HR Oh JW Kim KR Kim MJ Kim BJ. Chamber and field studies demonstrate differential amb a 1 contents in common ragweed depending on CO2 levels. Allergy, Asthma Immunol Res. (2018) 10:278–82. 10.4168/aair.2018.10.3.278 29676075 PMC5911447

[73] Albertine JM Manning WJ Da Costa M Stinson KA Muilenberg ML Rogers CA. Projected carbon dioxide to increase grass pollen and allergen exposure despite higher ozone levels. PLoS ONE. (2014) 9:1–6. 10.1371/journal.pone.0111712 25372614 PMC4221106

[74] Rogerieux F Godfrin D Sénéchal H Motta AC Marlière M Peltre G. Modifications of Phleum pratense grass pollen allergens following artificial exposure to gaseous air pollutants (O3, NO2, SO2). Int Arch Allergy Immunol. (2007) 143:127–34. 10.1159/000099079 17259730

[75] Abdelaal KA EL-Maghraby LM Elansary H Hafez YM Ibrahim EI El-Banna M. Treatment of sweet pepper with stress tolerance-inducing compounds alleviates salinity stress oxidative damage by mediating the physio-biochemical activities and antioxidant systems. Agronomy. (2020) 10:1–15. 10.3390/agronomy10010026

[76] Ahanger MA Mir RA Alyemeni MN Ahmad P. Combined effects of brassinosteroid and kinetin mitigates salinity stress in tomato through the modulation of antioxidant and osmolyte metabolism. Plant Physiol Biochem. (2020) 147:31–42. 10.1016/j.plaphy.2019.12.007 31838316

[77] Hasanuzzaman M Alam MM Rahman A Hasanuzzaman M Nahar K Fujita M. Exogenous proline and glycine betaine mediated upregulation of antioxidant defense and glyoxalase systems provides better protection against salt-induced oxidative stress in two rice (Oryza sativa L.) varieties. Biomed Res Int. (2014) 2014:1–17. 10.1155/2014/757219 24991566 PMC4065706

[78] Liu J Hasanuzzaman M Wen H Zhang J Peng T Sun H. High temperature and drought stress cause abscisic acid and reactive oxygen species accumulation and suppress seed germination growth in rice. Protoplasma. (2019) 256:1217–27. 10.1007/s00709-019-01354-6 31001689

[79] Saha I De AK Sarkar B Ghosh A Dey N Adak MK. Cellular response of oxidative stress when sub1A QTL of rice receives water deficit stress. Plant Sci Today. (2018) 5:84–94. 10.14719/pst.2018.5.3.387

[80] Satish L Rency AS Ramesh M. Spermidine sprays alleviate the water deficit-induced oxidative stress in finger millet (Eleusine coracana L. *Gaertn*.) plants. Biotech. (2018) 8:1–11. 10.1007/s13205-018-1097-2 29354374 PMC5764883

[81] Hasanuzzaman M Nahar K Gill SS Fujita M. Drought stress responses in plants, oxidative stress, antioxidant defense. In: Tuteja NS Gill S, editors. Climate Change Plant Abiotic Stress Tolerance. Weinheim: Wiley-VCH Verlag GmbH & Co. KGaA. p. 209–50.

[82] Hasanuzzaman M Bhuyan MHMB Zulfiqar F Raza A Mohsin SM Al Mahmud J. Reactive oxygen species and antioxidant defense in plants under abiotic stress: revisiting the crucial role of a universal defense regulator. Antioxidants. (2020) 9:1–52. 10.3390/antiox9080681 32751256 PMC7465626

[83] Bacsi A Dharajiya N Choudhury BK Sur S Boldogh I. Effect of pollen-mediated oxidative stress on immediate hypersensitivity reactions and late-phase inflammation in allergic conjunctivitis. J Allergy Clin Immunol. (2005) 116:836–43. 10.1016/j.jaci.2005.06.002 16210058 PMC3030477

