# Supplementary material for: Plant Stress Scenarios Differentially Affect Expression and IgE Reactivity of Grass Group-1 Allergen (β-Expansin) in Maize and Rice Pollen
Source: Front Allergy. 2022 Feb 10;3:807387. doi: 10.3389/falgy.2022.807387 (PMC8974862; doi:10.3389/falgy.2022.807387)
Supplement: Supplementary file 1 [file Data_Sheet_1.PDF]

Supplementary Material

Plant stress scenarios differentially affect expression and IgE reactivity of grass group-1 allergen (β-expansin) in maize and rice pollen

Yotin Juprasong[1,2,3] and Wisuwat Songnuan[2,3,4*]

[1]Graduate Program in Toxicology, Faculty of Science, Mahidol University, Bangkok, Thailand

[2]Center of Excellence on Environmental Health and Toxicology, Faculty of Science, Mahidol University, Bangkok, Thailand

[3]Systems Biology of Diseases Research Unit, Faculty of Science, Mahidol University, Bangkok, Thailand

[4]Department of Plant Science, Faculty of Science, Mahidol University, Bangkok, Thailand

* Correspondence:
Wisuwat Songnuan
wisuwat.son@mahidol.edu

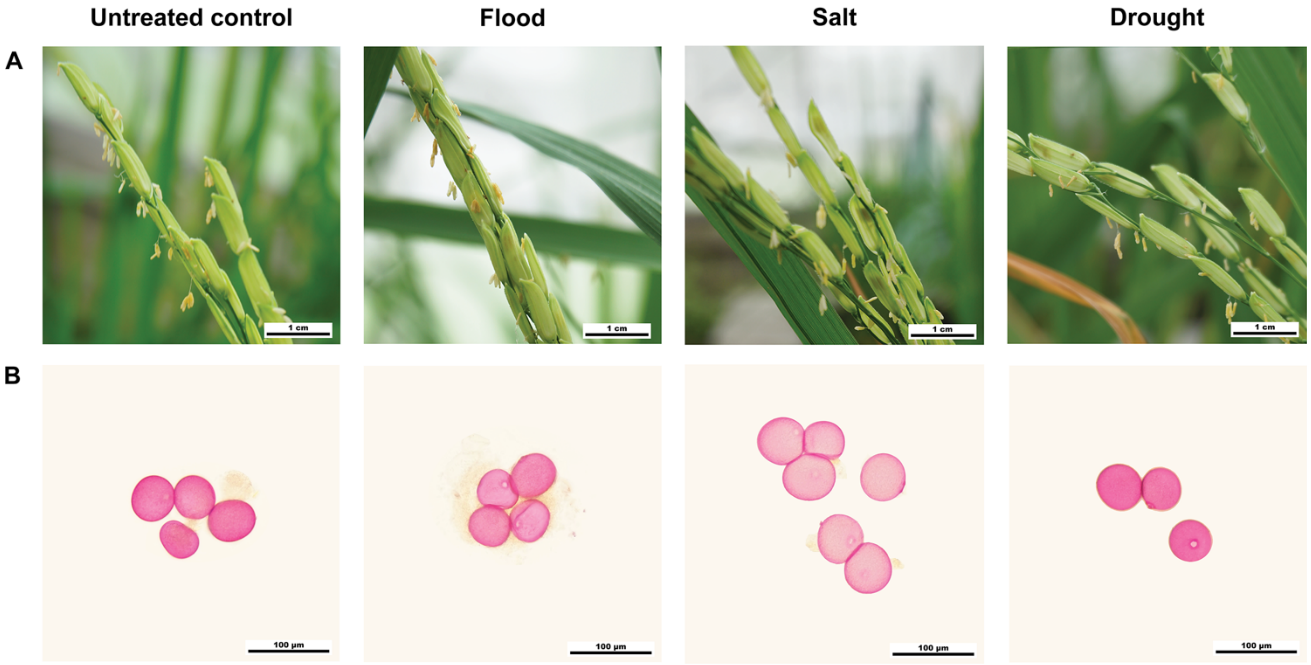

Figure S1. Inflorescences and pollen grains of experimental rice. Rice was treated with environmental-stress scenarios either untreated control (panel 1), flood (panel 2), salt (panel 3), or drought (panel 4). Rice inflorescences containing anthers were seen at day 7 (the reference scale: 1 cm) **(A)**. Pollen grains collected from each treatment were studied under a compound light microscope at the magnification of 40x (the reference scale of 100 μm) **(B)**.

Table S1. Demographic data and skin prick test (SPT) result of individual patient (no. 1-4) and control (no. 5-6) subjects.

Demographic data and SPT result	Subject number					
	1	2	3	4	5	6
1. Demographic data						
Gender	Female	Female	Female	Female	Male	Female
Age (year)	34	54	28	22	37	24
Smoking status	Yes	No	No	No	No	No
Pet	Yes	No	Yes	No	No	No
Family history with allergic disease	Yes	Yes	No	No	No	No
Medication[†]	Yes	Yes	Yes	Yes	No	No
2. SPT result: wheal size (width x length, cm) after tested with each allergen extract						
Bermuda grass	0[††]	3 x 4	8 x 10	6 x 8	0[††]	0[††]
Johnson grass	4 x 4	2 x 3[††]	8 x 9	9 x 8	0[††]	0[††]
Para grass	5 x 8	3 x 4	13 x 19	8 x 8	0[††]	0[††]

[†]Current medication with antihistamine and/or intranasal corticosteroid; [††]Negative SPT result
